# Supplementary material for: The role of additive and diffusive coupling on the dynamics of neural populations
Source: Sci Rep. 2023 Mar 13;13:4115. doi: 10.1038/s41598-023-30172-3 (PMC10011566; doi:10.1038/s41598-023-30172-3)
Supplement: Supplementary file 1 — Supplementary Information. [file 41598_2023_30172_MOESM1_ESM.pdf]

Supplementary Material

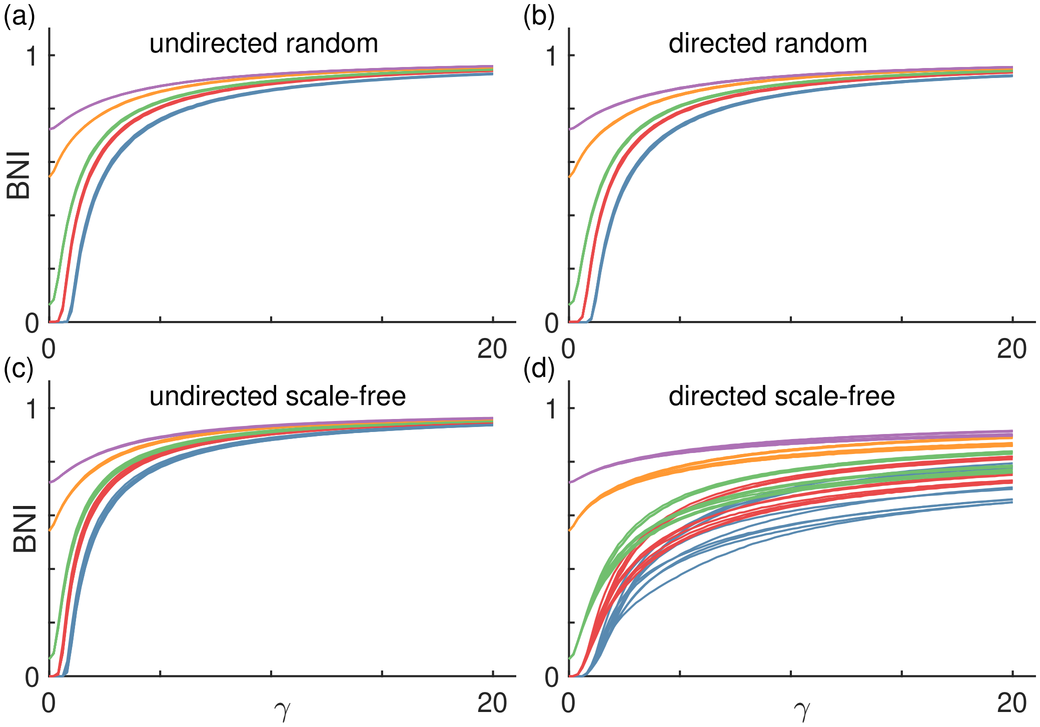

Figure S1. BNI as a function of γ using the additive coupling. As in Fig. 4, each panel shows BNI curves for different network topologies: (a) undirected random networks, (b) directed random networks, (c) undirected scale-free networks, and (d) directed scale-free networks. Each color corresponds to a different level of noise α: blue is $\alpha = 0.001$, red is $\alpha = 0.005$, green is $\alpha = 0.01$, orange is $\alpha = 0.03$, and purple is $\alpha = 0.05$. Finally, each curve corresponds to a different network realisation. We used 10 network realisations per network topology. The mean degree of all networks is $c = 8$.

Supplementary Text: Theta model

In the main text we compare additive, diffusive and mixed couplings within the bi-stable model presented in the Methods. Here, we expand our comparison to a different model of ictogenicity, the theta model[14,23,31].

The theta model can be used as a phenomenological BNM of seizure dynamics[14]. The phase θ_k characterises the activity at node k and it is described by the differential equation,

$$\dot{\theta}_k = (1 - \cos(\theta_k)) + (1 + \cos(\theta_k))I_k(t), \tag{S1}$$

where $I_k(t)$ is an input current to the node. At $I_k < 0$, the node is at a fixed stable phase $\theta_k^{(s)}$ (a 'resting state'). At $I_k = 0$, there is a saddle-node on invariant circle (SNIC) bifurcation. At $I_k > 0$, the node oscillates (the 'seizure state'). The current $I_k(t)$ is defined by

$$I_k(t) = I_0 + \xi_k(t) + \frac{1}{N}\sum_{j\neq k} A_{jk} G(\theta_k, \theta_j, \theta_k^{(s)}), \tag{S2}$$

where I_0 is the excitability of node k, $\xi_k(t)$ are noisy inputs, and the third term on the right hand side is the coupling term. N is the number of nodes, A_{jk} is the adjacency matrix, and $G(\theta_k, \theta_j, \theta_k^{(s)})$ is the coupling function. We consider two coupling functions:

$$G^a(\theta_k, \theta_j, \theta_k^{(s)}) = \gamma[1 - \cos(\theta_j - \theta_k^{(s)})], \tag{S3}$$

$$G^d(\theta_k, \theta_j, \theta_k^{(s)}) = \beta \sin\left(\frac{\theta_j - \theta_k}{2}\right), \tag{S4}$$

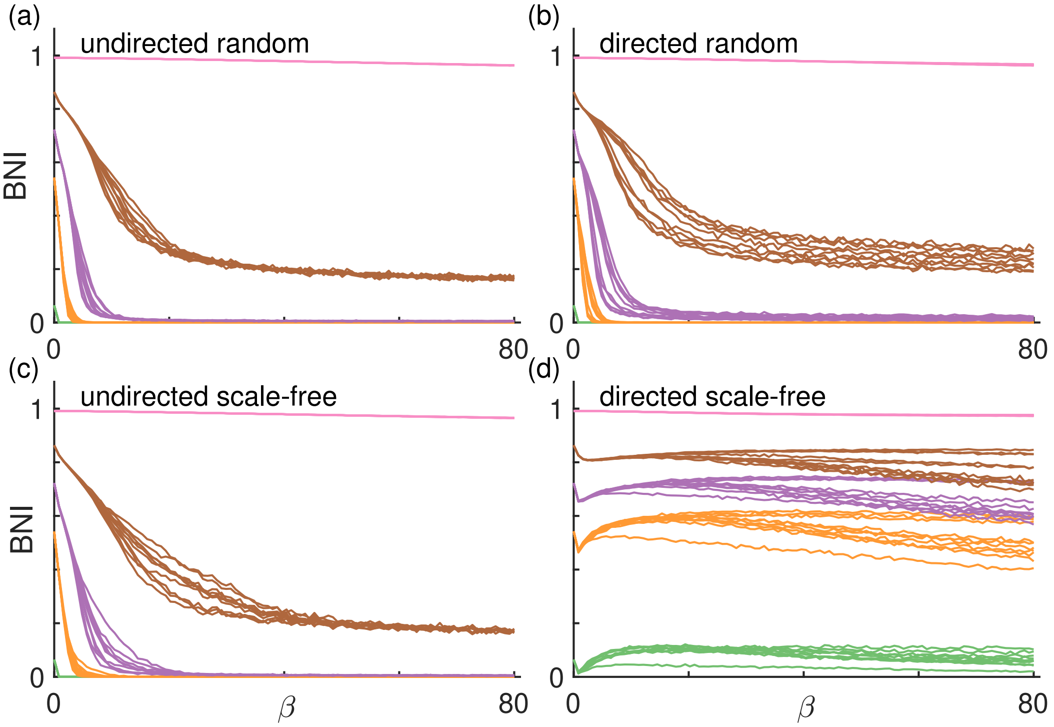

Figure S2. BNI as a function of β using the diffusive coupling. As in Fig. 5, each panel shows BNI curves for different network topologies: (a) undirected random networks, (b) directed random networks, (c) undirected scale-free networks, and (d) directed scale-free networks. Each color corresponds to a different level of noise α: green is $\alpha = 0.01$, orange is $\alpha = 0.03$, purple is $\alpha = 0.05$, brown is $\alpha = 0.1$, and pink is $\alpha = 1$. Finally, each curve corresponds to a different network realisation. We used 10 network realisations per network topology. The mean degree of all networks is $c = 8$.

where G^a corresponds to additive coupling, and G^d to diffusive coupling. G^a has been considered in the literature[14,23,31,34], whereas G^d has not.

The phase $\theta_k^{(s)}$ is a stable fixed point obtained from $\dot{\theta}_k = 0$ in the absence of noise and node interactions ($\xi^{(k)}(t) = 0$ and $\gamma = \beta = 0$),

$$\theta_k^{(s)} = -\Re\left\{\cos^{-1}\left(\frac{1+I_0}{1-I_0}\right)\right\}. \tag{S5}$$

At $I_0 > 0$ there is no stable fixed point and we use $\theta_k^{(s)} = 0$.

The noise term is independent across nodes and it is Gaussian distributed with zero mean and variance σ^2. Following previous studies[14,23,31,34], we use $I_0 = -1.2$ and $\sigma = 0.6$ in the case of the additive coupling. We consider $\sigma = 1.44$ in the case of the diffusive coupling. As in the bi-stable model, we had to consider a higher level of noise in the diffusive coupling than in the additive coupling because otherwise the network would always be in the resting state regardless of β.

To compare additive and diffusive couplings using this model, we computed the BNI and NI as we did in the main text for the bi-stable model. We also used the same networks (see Section 2.3) so that to enable comparisons between the theta and bi-stable models. We integrated the stochastic equations (S1) using the Euler-Maruyama method with step size $h = 10^{-2}$.

In the theta model, the BNI is defined as follows

$$\text{BNI} = \frac{1}{N}\sum_{k=1}^{N}\frac{t_{sz}^{(k)}}{M'}, \tag{S6}$$

where $t_{sz}^{(k)}$ is the time that node k spends in the seizure state during a total simulation time M'. We used $M' = 4\times 10^6$ as in Ref.[23,31]; see Lopes *et al.*[14] for more details on the calculation of $t_{sz}^{(k)}$. The NI is the same as presented in the main text.

Figures S6 and S7 show the BNI as function of γ and β for the additive and diffusive couplings respectively. The results are similar to those observed within the bi-stable model. Namely, the BNI grows monotonically with γ and decreases with β.

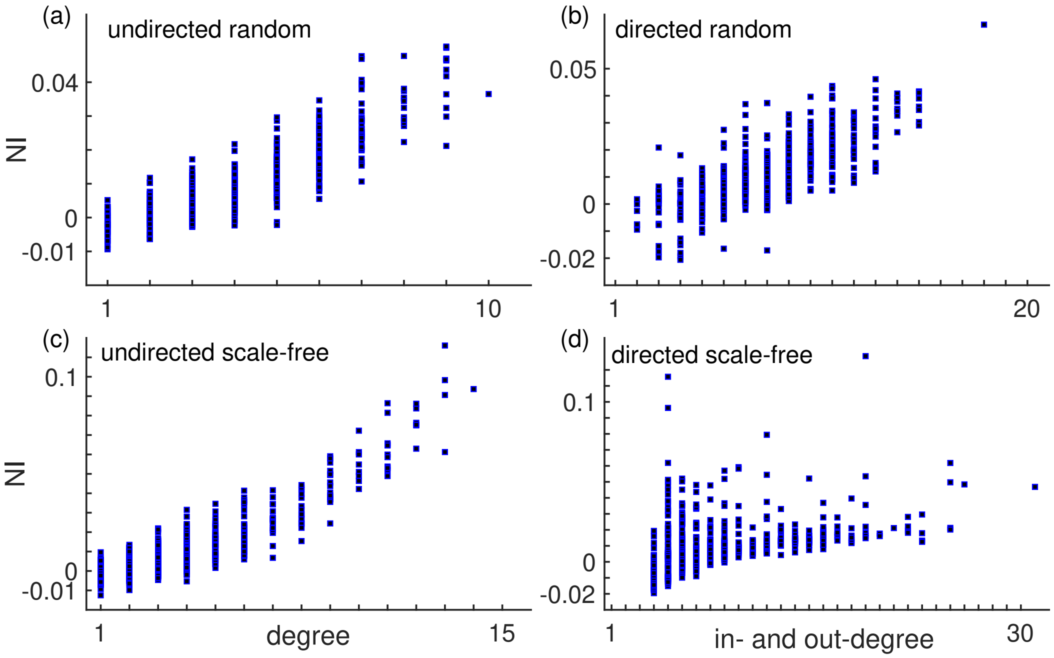

Figure S3. NI versus degree across different network topologies using the additive coupling. Each panel corresponds to a different network topology: (a) undirected random, (b) directed random, (c) undirected scale-free, and (d) directed scale-free networks. In the case of the directed networks, the horizontal axis shows the sum of in- and out-degree. This figure combines the NI distributions across 10 different network realisations per network topology. The average correlation between NI and degree is 0.75 and the average p-value is 0.0015. All parameters are the same as in Fig. 7.

Also, increasing the mean degree of the networks has a similar effect to increasing γ or β. A difference between these results and those observed with the bi-stable model is that in the case of the diffusive coupling in directed networks, we found a local minimum in some BNI curves in the bi-stable model (see Figs. 5 and S2) but not in the theta model. However, we have not performed an exhaustive parameter search for such behaviour in the theta model, therefore we cannot exclude the possibility that it may exist for other parameters.

Figure S8 compares NI distributions computed with additive and diffusive couplings in the theta model. The results are qualitatively the same as those found in the bi-stable model (see Fig. 7). Nodes with higher (positive) NI in the additive coupling case are the nodes with lowest (negative) NI in the diffusive coupling case. Furthermore, the NI in the additive coupling has higher variability than in the diffusive coupling. Figures S9, S10, and S11 are also in agreement with the corresponding in the bi-stable model. Briefly, the NI correlates (anti-correlates) with node degree in the additive (diffusive) coupling (except when using additive coupling in directed scale-free networks). The weighted Kendall correlation rank τ is close to -1 in all networks except directed scale-free networks, showing that the NI from additive and diffusive couplings rank nodes in reverse order. The directed scale-free networks is presumably an exception due to its highly heterogeneous nature in terms of degree distribution. We speculate that the role of in-degree and out-degree is different in the two coupling cases, but not the 'reverse', as it seems to be the case when considering undirected networks, where the in-degree is equal to out-degree.

Finally, Fig. S12 compares the NI distributions between the theta and bi-stable models when using the same coupling function. We observe a high level of agreement in all networks, particularly when using the additive coupling. The agreement is lower in the diffusive coupling presumably because in this case the variability in NI is lower than in the additive coupling and therefore there is a higher chance of some node orderings being random.

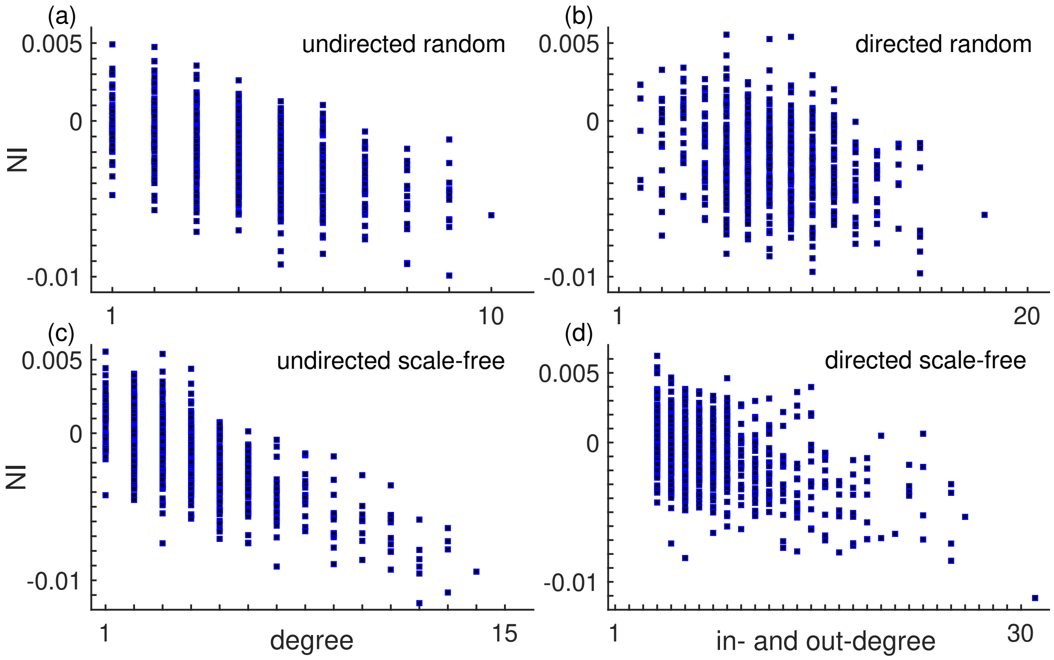

Figure S4. NI versus degree across different network topologies using the diffusive coupling. Each panel corresponds to a different network topology: (a) undirected random, (b) directed random, (c) undirected scale-free, and (d) directed scale-free networks. In the case of the directed networks, the horizontal axis shows the sum of in- and out-degree. This figure combines the NI distributions across 10 different network realisations per network topology. The average correlation between NI and degree is -0.57 and the average p-value is 0.0017. All parameters are the same as in Fig. 7.

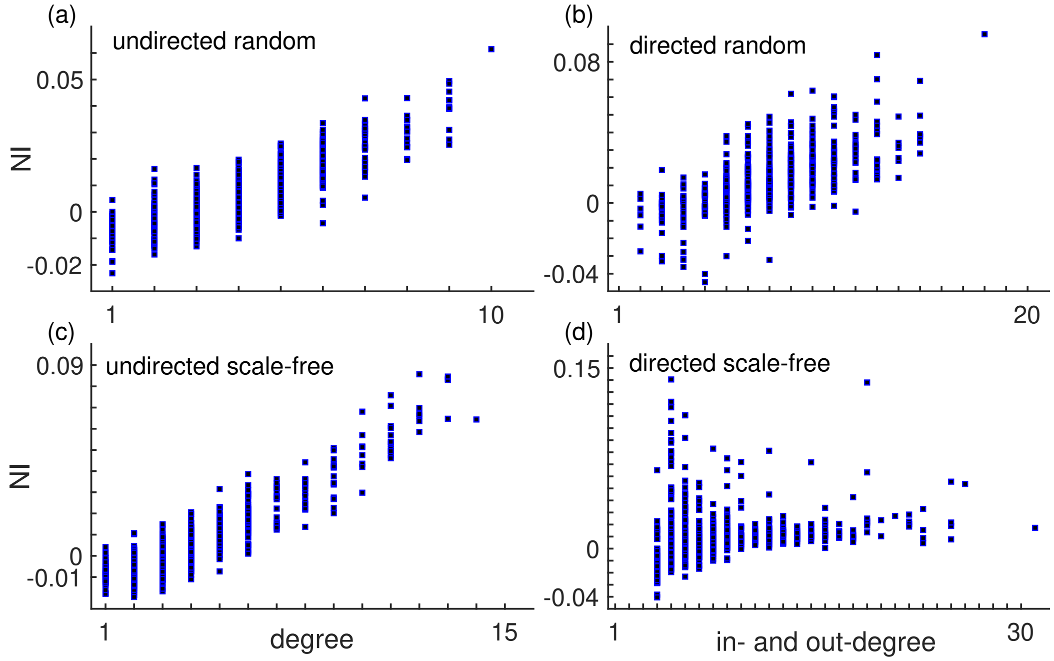

Figure S5. NI versus degree across different network topologies using the mixed coupling. Each panel corresponds to a different network topology: (a) undirected random, (b) directed random, (c) undirected scale-free, and (d) directed scale-free networks. In the case of the directed networks, the horizontal axis shows the sum of in- and out-degree. This figure combines the NI distributions across 10 different network realisations per network topology. The average correlation between NI and degree is 0.69 and the average p-value is 0.040. All parameters are the same as in Fig. 7.

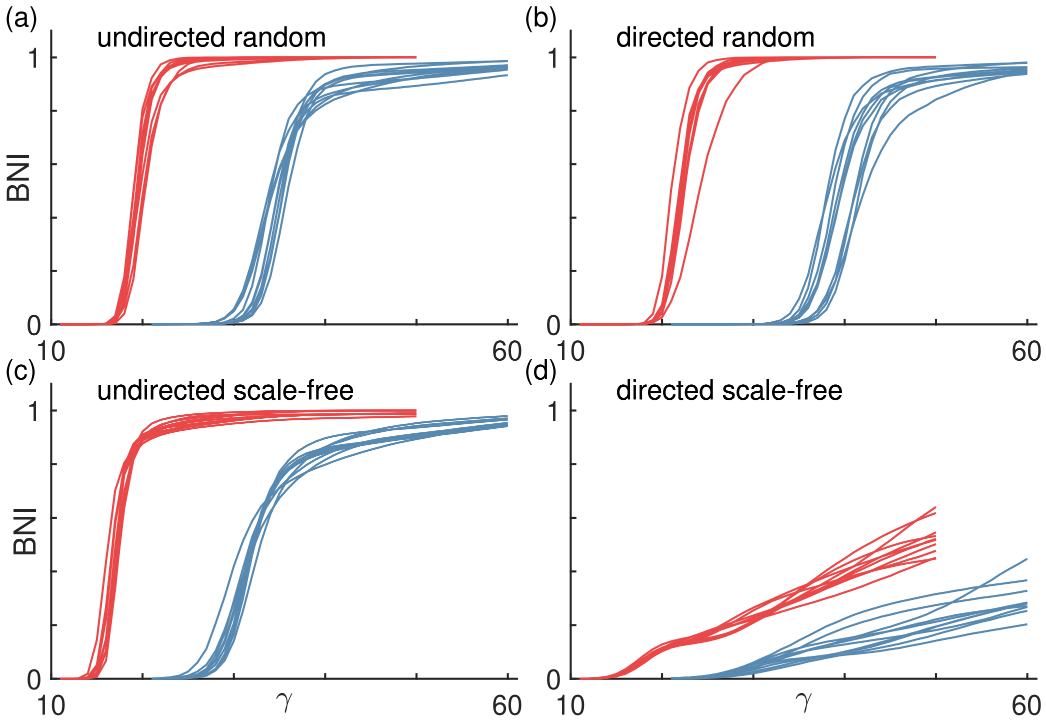

Figure S6. BNI as a function of γ using the additive coupling and the theta model. Each panel shows BNI curves for different network topologies: (a) undirected random networks, (b) directed random networks, (c) undirected scale-free networks, and (d) directed scale-free networks. Curves in blue correspond to networks with mean degree $c = 4$, and curves in red correspond to networks with $c = 8$. Each curve corresponds to a different network realisation. We used 10 network realisations per network topology.

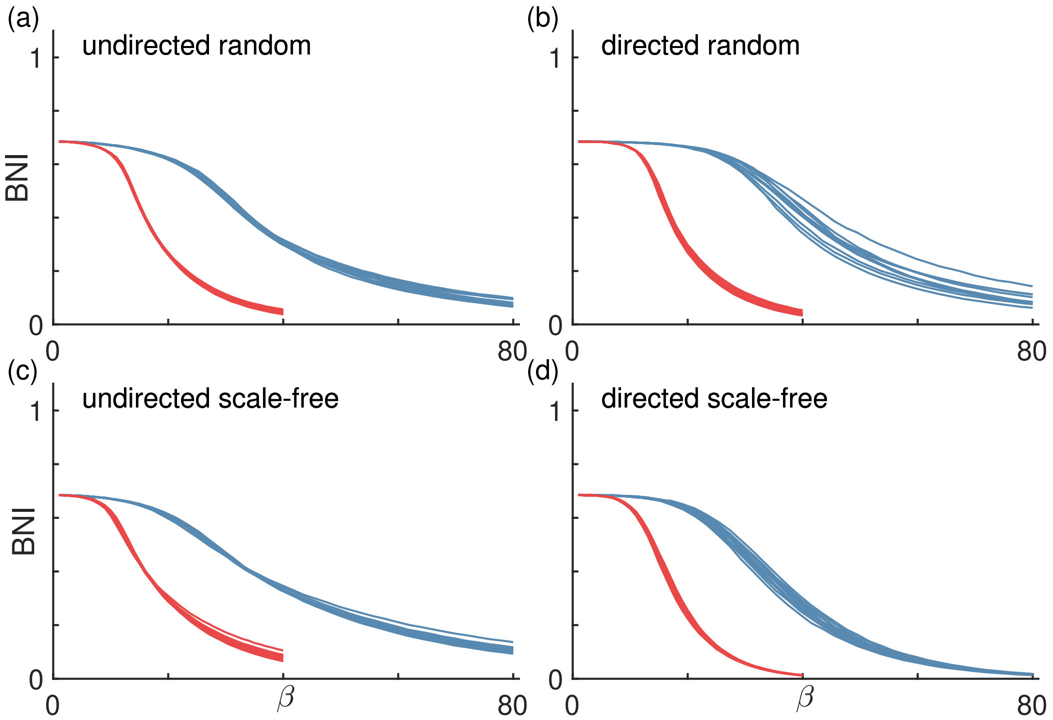

Figure S7. BNI as a function of β using the diffusive coupling and the theta model. Each panel shows BNI curves for different network topologies: (a) undirected random networks, (b) directed random networks, (c) undirected scale-free networks, and (d) directed scale-free networks. Curves in blue correspond to networks with mean degree $c = 4$, and curves in red correspond to networks with $c = 8$ (we used a different range of β for the two sets of curves). Each curve corresponds to a different network realisation. We used 10 network realisations per network topology.

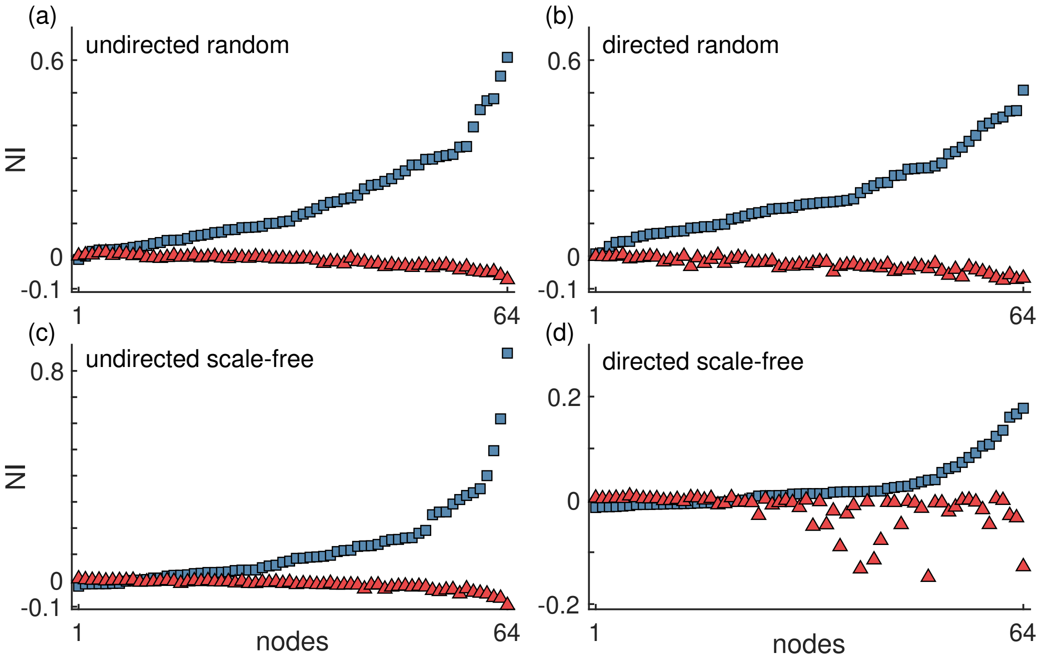

Figure S8. Representative NI distributions of (a) undirected random, (b) directed random, (c) undirected scale-free, and (d) directed scale-free networks using the additive and diffusive couplings. The blue squares represent the NI computed using the additive coupling and the red triangles correspond to the diffusive coupling. The nodes were sorted such that the NI grows monotonically for the additive coupling. The error bars are smaller than the symbols. The parameters γ and β were chosen such that $\mathrm{BNI}_{\mathrm{pre}} = 0.5$. All networks had mean degree $c = 4$.

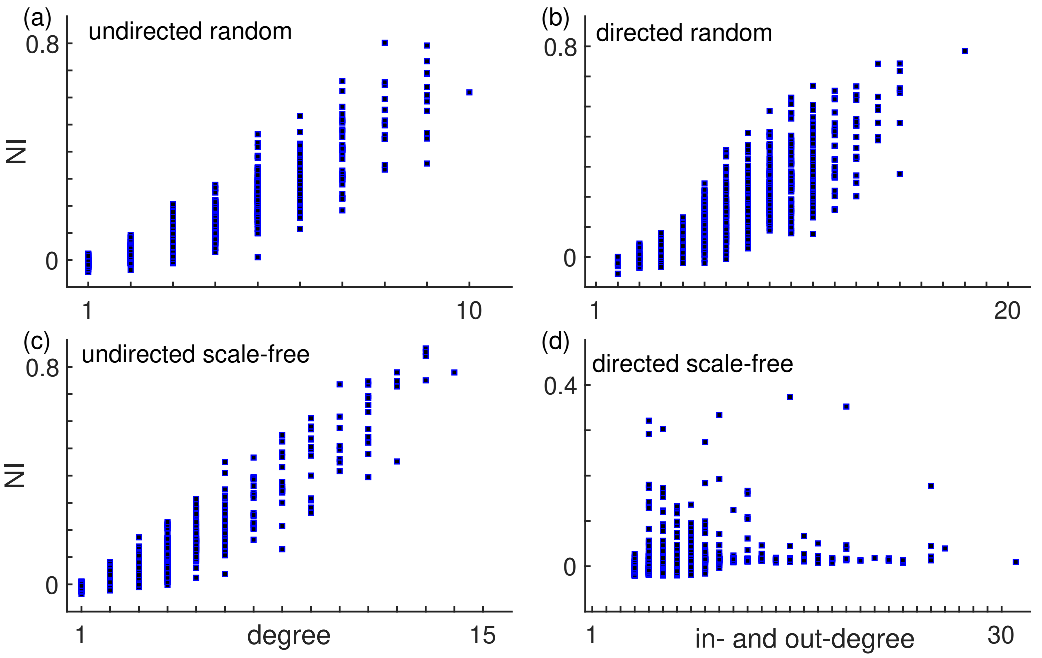

Figure S9. NI versus degree across different network topologies using the additive coupling and the theta model. Each panel corresponds to a different network topology: (a) undirected random, (b) directed random, (c) undirected scale-free, and (d) directed scale-free networks. In the case of the directed networks, the horizontal axis shows the sum of in- and out-degree. This figure combines the NI distributions across 10 different network realisations per network topology. The average correlation between NI and degree is 0.73 and the average p-value is 0.055. We used networks with $c = 4$.

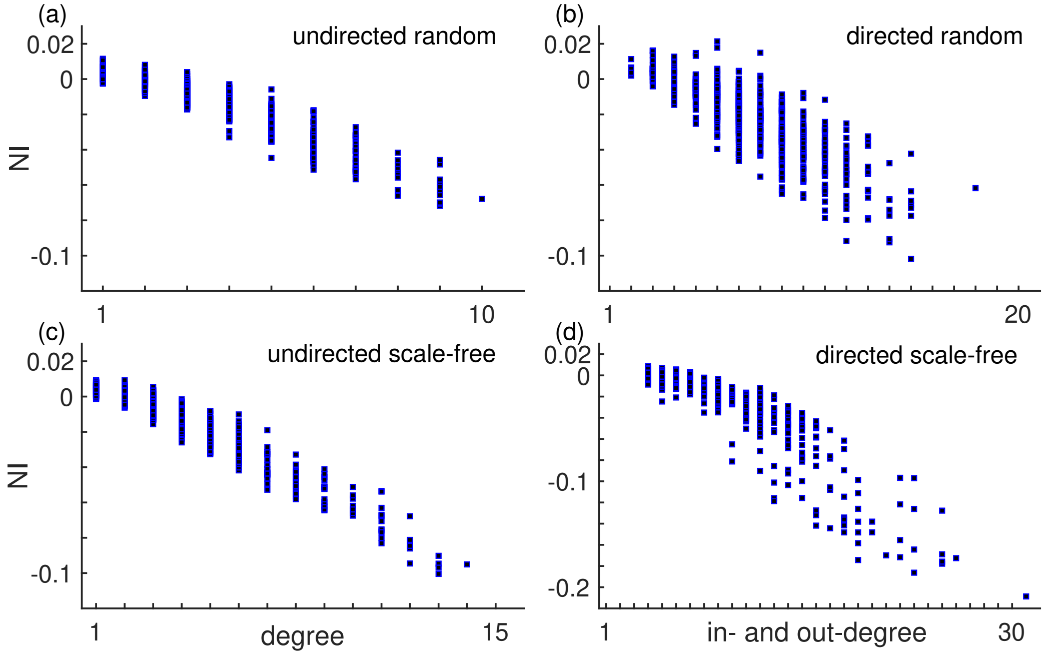

Figure S10. NI versus degree across different network topologies using the diffusive coupling and the theta model. Each panel corresponds to a different network topology: (a) undirected random, (b) directed random, (c) undirected scale-free, and (d) directed scale-free networks. In the case of the directed networks, the horizontal axis shows the sum of in- and out-degree. This figure combines the NI distributions across 10 different network realisations per network topology. The average correlation between NI and degree is -0.91 and the average p-value is 5.7×10^{-16}. We used networks with $c = 4$.

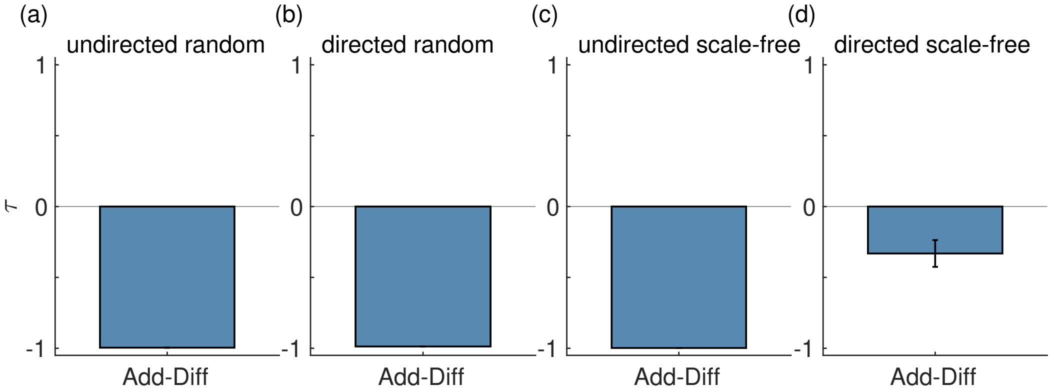

Figure S11. Comparison of NI distributions using the additive and diffusive couplings in the theta model. The weighted Kendall correlation rank τ quantifies the consistency of ordering nodes according to their NI values when using the different couplings. Different panels show the comparison between additive and diffusive coupling for different network topologies: (a) undirected random, (b) directed random, (c) undirected scale-free random, and (d) directed scale-free networks. The error bars represent the standard error across the 10 network realisations per network topology. We considered networks with $c = 4$.

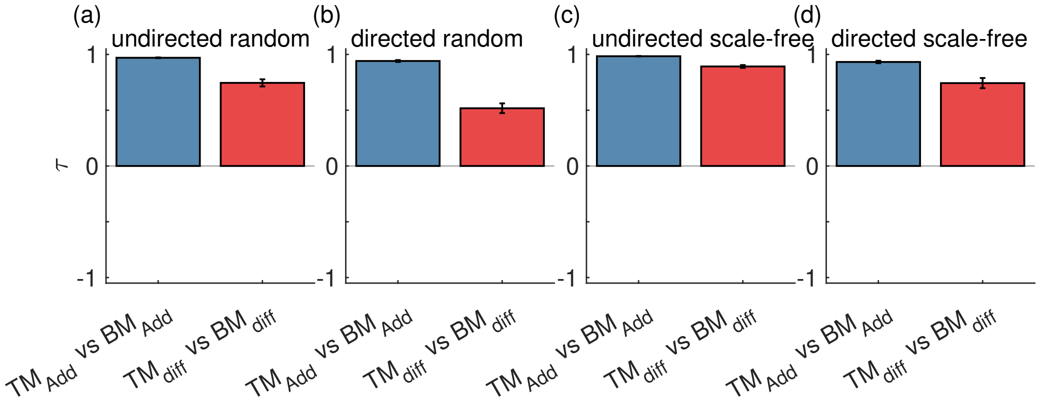

Figure S12. Comparison of NI distributions between the theta and bi-stable models using the additive and diffusive couplings. The blue (red) bars correspond to the comparison between the two models using both the additive (diffusive) coupling. Different panels show the comparison for different network topologies: (a) undirected random, (b) directed random, (c) undirected scale-free random, and (d) directed scale-free networks. The error bars represent the standard error across the 10 network realisations per network topology. We considered networks with $c = 4$.
